# Oncogenic Kit Triggers Shp2/Erk1/2 Pathway to Down-Regulate the Pro-Apoptotic Protein Bim and to Promote Apoptosis Resistance in Leukemic Cells

**DOI:** 10.1371/journal.pone.0049052

**Published:** 2012-11-07

**Authors:** Dorothée Selimoglu-Buet, Isabelle Gallais, Nicole Denis, Christel Guillouf, Françoise Moreau-Gachelin

**Affiliations:** Institut Curie, Inserm-U830, Paris, France; Institut National de la Santé et de la Recherche Médicale (INSERM), France

## Abstract

Oncogenic mutations leading to persistent kinase activities are implicated in various human malignancies. Thereby, signaling pathway-targeted therapies are powerful customized treatment to eradicate cancer cells. In murine and human leukemia cells harboring mutations in Kit, we previously showed that distinct and independent pathways controlled resistance to apoptosis or cell cycle. A treatment with PI3Kinase inhibitors to reduce cell proliferation combined with inhibitors of Erk1/2 activity to promote apoptosis had synergistic effects allowing eradication of leukemia cell growth. We reported here that Bim_EL_, a pro-apoptotic member of the Bcl2 family proteins, is the target of Erk1/2 signaling and that its down-regulation is responsible for the apoptosis resistance of murine and human leukemic cells. Downstream of Kit mutant, the tyrosine phosphatase Shp2 maintains Bim_EL_ expression at a low level, through Erk/2 activation and proteosomal Bim_EL_ degradation. This process is controlled by Shp2 independently of other signaling pathways activated downstream of oncogenic Kit, demonstrating that Shp2 is a key regulator of Bim expression in the context of an oncogenic signaling. The increase in Bim_EL_ expression is associated to an increased apoptosis. Moreover, the depletion of Bim overcomes apoptosis associated with Erk1/2 inactivation in UO126-treated leukemic cells, thereby establishing the contribution of Bim to drug-induced apoptosis. These data provide a molecular rationale for using BH3 mimetics in combination with PI3K inhibitors to treat leukemia, especially in the case of an oncogenic signaling refractory to Tyrosine Kinase inhibitors.

## Introduction

Oncogenic mutations in the receptor tyrosine kinase for stem cell factor (Kit) underly the development of a variety of neoplasms including leukemia. Activating Kit mutations generate many perturbations in various signaling networks and, thus, understanding their contribution to the malignant phenotype may provide a molecular rationale to design pathway-targeted therapies. This might be a key issue for patients with Kit-driven neoplasms that have a high probability of developing resistance to Tyrosine Kinase inhibitors.

In the *spi-1/PU.1*-transgenic mouse model, activating Kit mutations drive malignant transformation during the erythroleukemic process. In this model, the founding oncogenic event is the inappropriate overexpression of Spi-1/PU.1, a master transcriptional regulator of B lymphopoiesis and myelopoiesis that leads to inhibition of terminal erythroid differentiation and an extensive proliferation of proerythroblastic cells (preleukemic cells) [1]. Later on, the blastic crisis is associated with occurrence of somatic mutations in Kit that confer growth factor autonomy and tumorigenicity to proerythroblastic cells (leukemic cells: so-called HS2 cells) [2], [3]. The Kit mutations target residues D814 or D818 in the phosphotransferase domain, which are homologous to human residues D816 or D820 mutated in patients with mastocytosis or AML. Our studies on the biochemical networks connecting Kit mutant to leukemogenesis have demonstrated that independent signaling pathways contribute to malignancy: cell survival is driven by the Kit/Shp2/Ras/Mek/Erk1/2 pathway whereas G1/S transition during cell cycle is accelerated by both the Kit/Stat5 and Kit/PI3K/Akt pathways [4]. Importantly, eradication of leukemic cells was only achieved with the concomitant inhibition of cell cycle and survival. This allowed to define drugs combinations for leukemia therapy. Indeed, the combined use of two clinically relevant compounds NVP-BEZ235 [5], an inhibitor targeting all isoforms of the PI3-Kinase as well as mTOR and Obatoclax [6], an antagonist of pro-survival Bcl2 factors, had strong synergistic effects to eradicate the growth of leukemic cells [4].

The activity of the mitochondrial pathway that controls apoptosis results from the coordination of interacting pro-apoptotic and anti-apoptotic proteins of the Bcl2 family [7]. An imbalance in this interplay can lead to a survival advantage that promotes neoplasm development. For example, amplification of the *Mcl-1* gene is a frequent somatic event in human cancer [8] and genetic alterations that activate anti-apoptotic proteins of the Bcl2 family such as Bcl2 or Mcl-1 occur in hematopoietic malignancies [9]. Otherwise, somatic mutations in the pro-apoptotic factor Bax confer resistance to apoptosis in solid and hematopoietic tumors [10]. In addition, homozygous deletions of the pro-apoptotic *Bim* gene are identified in lymphoma [11].

Bim is a BH3-only protein of the Bcl2 family that is essential for apoptosis induced by growth-factor deprivation in a broad range of cell types [7]. Bim expression is subjected to different modes of regulation at both transcriptional and post-translational levels [12], that are governed by various signaling pathways, including the Erk1/2 and PI3K/Akt pathways [13].

In the present study, we searched whether proteins of the Bcl2 family are mediators of the apoptosis resistance of leukemic cells. We show that Erk1/2 activation induces the phosphorylation and the down-regulation of Bim_EL_ via proteasomal degradation. This process was controlled by the Tyrosine phosphatase Shp2 independently of other signaling activated by Kit mutant showing that Shp2 as an important regulator of Bim expression in the context of an oncogenic signaling. The increase in Bim_EL_ expression was associated with an increased apoptosis and the depletion of Bim overcomes apoptosis associated with Erk1/2 inactivation demonstrating that Bim_EL_ is a crucial mediator of apoptosis resistance in leukemic cells. These functions of Bim_EL_ make relevant the association of BH3 mimetics with PI3K inhibitors to eradicate the leukemic growth and provide a molecular basis to improve the response obtained with the combined inhibition of signals controlling survival and cell cycle.

## Materials and Methods

### Cell Lines and Inhibitors

HS2 cell lines were established from the spleen of leukemic *spi-1*-transgenic mice as previously described [1]. The human mast cell leukemia subclone HMC-1.2 [2], [3] harboring Kit^V560G^ and Kit^D816V^ was kindly provided by Dr JH Butterfield (Mayo Clinic, Rochester, MN). Murine and human cells were cultured in alpha minimun essential medium (αMEM, GibcoBRL) supplemented with 10% fetal calf serum (FCS, GibcoBRL).

For signaling inhibition, cells were treated for 4 h with the Kit inhibitors PP1 (Enzo life science), PP2 (Calbiochem) and Imatinib mesilate (Novartis-Pharma), the PI3-kinase inhibitor NVP-BEZ235 (Novartis Pharma) and the MEK inhibitor UO126 (Promega). For proteasome inhibition, cells were treated for 2 h or 4 h with MG132 (Calbiochem).

### Lentiviral and Retroviral Vector Production

pLKO.1-Puro vectors transducing shRNA targeting Shp2 (Shp2-shRNA77 or Shp2-shRNA78), a control non-silencing scramble shRNA (ns) or shRNA targeting Bim (Bim-shRNA92 or Bim-shRNA94) were purchased from Mission shRNA lentivirus library (Sigma-Aldrich). Lentiviral particles were generated as previously described [14]. Freshly prepared supernatants were used to transduce HS2 cells. 48 h after the start of infection, infected HS2 cells were selected for puromycin (1 µg/mL- Cayla, Toulouse, France) resistance as previously described [4].

Knocking-down of Stat5 in 606HS2 and 931HS2 cells was performed through cell infection with a lentiviral vector encoding GFP as reporter gene and transducing Stat5-shRNA17 or control ns-shRNA. 48 h after the start of infection, infected HS2 cells were sorted for GFP expression, using a FACSVantage (Becton Dickinson), as previously described [4]. A lentiviral vector encoding an anti-luciferase shRNA was used as control.

Overexpression of Shp2 in 606HS2 and 931HS2 cells was performed through infection with retroviruses transducing the wild-type Shp2-MT or the Shp2^C459S^-MT mutant and then selected for puromycin resistance. The cDNA encoding murine Shp2^WT^ was cloned in frame with a tag-myc epitope in C-terminus into pMSCV retroviral vector (Shp2^WT^-MT). The Shp2^C459S^ mutant (Shp2^ C459S^-MT) was generated by mutagenesis of the wild-type Shp2 cDNA using the QuichChange Site-Directed Mutagenesis System (Stratagene, LaJolla, CA). The empty vector (c) was used as control.

### Cell Death and Cell Apoptosis

The percentage of dead cells was evaluated by trypan blue exclusion using a Vi-Cell analyzer (Beckman Coulter). The percentage of apoptotic cells was analyzed by labeling the cells with cleaved caspase3-antibody. Cells were fixed with PFA1%, permeabilized with 70% cold ethanol and stained with phycoerythrin(FITC)-conjugated anti-active caspase3 antibodies (Becton Dickinson). Flow cytometry was performed with FACSsort (Becton Dickinson) and data were analyzed using Flow Jo (Treestar).

### Immunoblotting and Antibodies

Whole cell extracts were fractionated by SDS-PAGE and immunoblotted as previously described [15]. Antibodies raised against the phosphorylated forms of Akt(S473) (# 4060S), Erk1/Erk2(Thr202-Tyr204) (# 9106S), the cleaved form of caspase-3 (# 9661S) and the constitutive forms of Bim (# 2819), Bcl-x_L_ (# 2764), Bak (# 3814), Akt (# 4685) and Erk1/Erk2 (# 9102S) were all from Cell Signaling. Anti-Stat5 (# SC836) and anti-Bax (# SC493) antibodies were from Santa Cruz biotechnology, anti-Bim (Ser65) antibody from Upstate (# 36-004), anti-Mcl1 antibody from Rockland Immunochemicals (# 600-401-394), anti-Shp2 antibody from BD Transduction Laboratories™ (# 610621) and anti-βactin antibody from Sigma-Aldrich (# A5441).

## Results and Discussion

### Bim_EL_ is the Pro-apoptotic Factor Down-regulated by Erk1/2 Activity in Leukemic Cells

**Figure 1:**
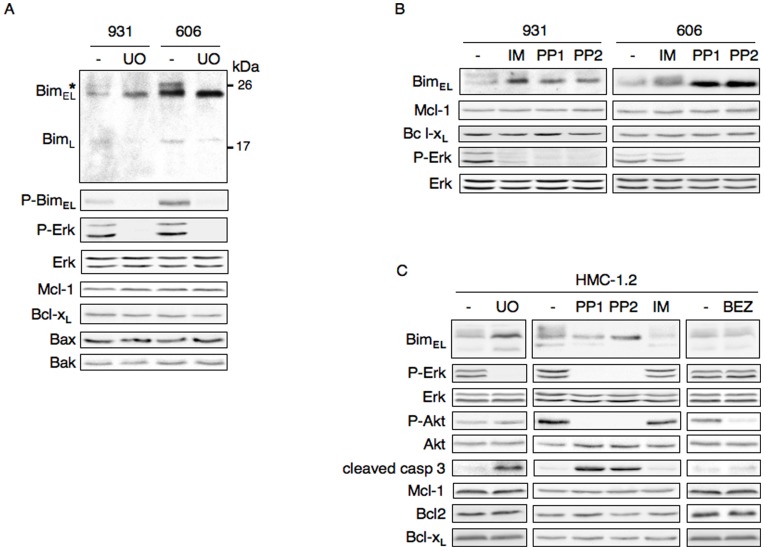
The down-regulation of Bim protein occurs downstream of the activation of Erk1/2 and Kit mutant in Kit-driven leukemic cells. (A) Whole cell extracts from 606HS2 and 931HS2 cells treated or not (–) for 4 h with the Mek inhibitor UO126 (10 µM) were fractionated by SDS-PAGE and immunoblotted using antibodies raised against the phosphorylated forms of Erk1/Erk2(Thr202-Tyr204) and Bim (Ser65) and the constitutive form of Bim, Erk1/Erk2, Mcl1 and Bcl_XL_,. *indicate the band corresponding to the phosphorylated form of Bim_EL_. Molecular weight standards are indicated on the right. (B) Whole cell extracts from 606HS2 and 931HS2 cells treated or not (–) for 4 h with Kit inhibitors PP1 (4 µM), PP2 (4 µM) and Imatinib Mesilate (IM: 0.75 µM) were immunoblotted using indicated antibodies. Data are a representative experiment out of three. (C) Whole cell extracts from human HMC-1.2 cells treated or not (–) for 4 h with Mek inhibitor UO126 (15 µM) or Kit inhibitors IM (10 µM), PP1 (10 µM) and PP2 (10 µM) or PI3K inhibitor NVP-BEZ235 (25 nM) were analyzed by immunoblotting using indicated antibodies. Data are a representative experiment out of three.

We have recently shown that apoptosis resistance of HS2 leukemic cells is induced by the activation of the Erk1/2 pathway [4]. To go further we investigated which mediators of the intrinsic apoptotic pathway were targeted by Erk1/2 activity. In diverse cell types, activation of Erk1/2 signaling can lead either to the up-regulation of pro-survival proteins of the Bcl-2 family, notably Bcl-2, Bcl-x_L_ and Mcl-1, or to the decrease or inactivation of pro-apoptotic proteins such as Bim_EL_ to achieve cell survival [13]. We analyzed the expression of the pro-survival factors Bcl-2, Bcl-x_L_, Mcl-1, the apoptotic mediators Bax, Bak and Bim in HS2 leukemic cells in response to a treament with the Mek inhibitor UO126 as an apoptosis inducer. Immunoblot analyses showed that expression levels of Mcl-1, Bcl-x_L_, Bax and Bak were not changed in cells treated with UO126 compared with untreated cells (Figure 1A). Bcl-2 expression was not detectable in HS2 cells (data not shown). Bim is expressed as three major isoforms generated by alternative splicing: a short, long and extra-long protein named Bim_S_, Bim_L_ and Bim_EL_ [16]. In HS2 cells, Bim_EL_ was the most abundant isoform expressed as two bands, expression of Bim_L_ was modest while Bim_S_ was undetectable. The treatment of HS2 cells with UO126 was associated with an increased expression of the lower band of Bim_EL_. Erk1/2 phosphorylates some serine residues on Bim_EL_ [17], [18] and an antibody that is specific for Bim_EL_ phosphorylated at Ser^65^ recognizes the Bim_EL_ upper band. This phosphorylated form was undetectable when HS2 cells were treated with UO126 whereas the total amount of Bim_EL_ was increased (Figure 1A). Thus, both phosphorylation and expression level of Bim_EL_ were dependent on the activity of Erk1/2.

Given that Erk1/2 activation depends on Kit mutant activity in leukemic cells, we controlled that Bim_EL_ expression was changed in response to the inhibition of Kit. The Kit^D818Y^ kinase activity in 931HS2 cells is sensitive to IM (Imatinib Mesylate or Gleevec®), PP1 and PP2 whereas Kit^D814Y^ kinase activity in 606 HS2 cells is sensitive to PP1 and PP2 but resistant to IM [15]. Treatment of 931HS2 cells with the Kit inhibitors IM, PP1 and PP2 abolished Bim_EL_ phosphorylation and promoted an increase in Bim_EL_ unphosphorylated form, a result comparable to that observed in response to UO126 treatment (Figure 1B). This treatment did not change Mcl-1 and Bcl-x_L_ expression. Treatment of 606HS2 cells with PP1 and PP2 but not with IM abolished Bim_EL_ phosphorylation as well as Erk1/2 activation and promoted an increase in Bim_EL_ unphosphorylated form. Thus, both phosphorylation and expression levels of Bim_EL_ depend on the activation of Erk1/2 downstream of Kit mutant in HS2 leukemic cells.

These results were confirmed with the human HMC-1.2 cell line derived from an AML patient with a mast cell leukemia. These cells express mutant Kit^D816V^ and the mutant Kit^D816^ in human, like mutant Kit^D814^ in mouse, promotes survival of leukemic cells through activation of the Kit/Shp2/Mek/Erk pathway [2], [4]. We analyzed in HMC-1.2 cells whether Bim phosphorylation was dependent on either Kit or Erk1/2 activities. In cells treated with PP1, PP2 or UO126, an increase in the expression of Bim_EL_ was observed when the Erk1/2 activation was abolished, whereas no change was detected in the expression of Bcl-2, Bcl-x_L_ and Mcl-1 (Figure 1C). In agreement with the resistance of Kit^D816V^ to IM [2], [4], neither expression of Bim_EL_ nor activation of Erk1/2 was modified by IM treatment. These results indicate that the ability of activated Erk1/2 to repress Bim expression downstream of Kit mutant is faithfull reproduced in human cell line.

### The Tyrosine Phosphatase Shp2 Controls the Down-regulation of Bim

**Figure 2:**
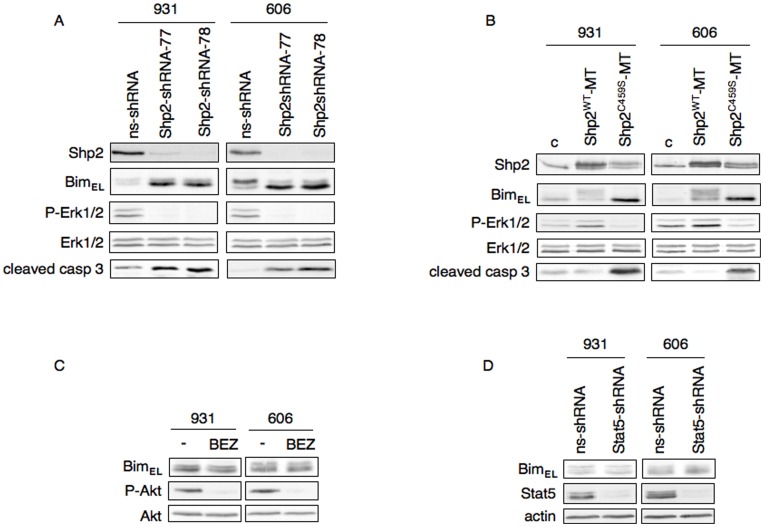
The tyrosine phosphatase Shp2 controls the down-regulation of Bim. (A) Knocking-down of Shp2 in 606HS2 and 931HS2 cells. Whole cell extracts were immunoblotted using antibodies raised against Shp2, P-Erk1/2, Erk1/2, Bim and the cleaved form of caspase-3. (B) Overexpression of Shp2 in 606HS2 and 931HS2 cells. Whole cell extracts were immunoblotted using indicated antibodies. The expression of Shp2^WT^-MT and Shp2^ C459S^-MT was detected as the band above the endogenous Shp2. (C) Whole cell extracts from 606HS2 and 931HS2 cells treated or not (–) for 4 h with PI3K inhibitor NVP-BEZ235 (120 nM) were immunoblotted using indicated antibodies. (D) Knocking-down of Stat5 in 606HS2 and 931HS2 cells. Whole cell extracts were immunoblotted using anti-Bim, anti-Stat5 antibodies and anti-β actin antibody as loading control.

Besides the Erk1/2 pathway, Stat5 and PI3K/Akt pathways are activated downstream of Kit mutant, all cooperating to the extensive proliferation of HS2 leukemic cells [4]. To clarify whether Bim expression was specifically dependent on Erk1/2 activity, we investigated whether Shp2 was a regulator of Bim expression. Indeed, our previous data have shown that Shp2 was the proximal effector of Kit mutant in activating Erk1/2 and mediating apoptosis resistance in leukemic cells [4]. We first examined Bim_EL_ expression in response to Shp2 knock-down by RNA interference. HS2 cells were infected with two lentiviral vectors transducing independent Shp2 short hairpin RNAs (shRNAs 77 or 78). Both shRNAs induced a major decrease (90–95%) in Shp2 protein level compared to a non silencing shRNA. Shp2 depletion abolished Erk1/2 activation and induced apoptosis as deduced from the detection of cleaved caspase-3 on immunoblotting (Figure 2A). Notably, Shp2 depletion was associated with an increase in the unphosphorylated form of Bim_EL_.

Conversely, we examined Bim_EL_ expression in response to Shp2 overexpression. HS2 cells were infected with a retrovirus transducing a Myc-tagged wild-type Shp2. The overexpression of wild-type Shp2 resulted in an increase in Erk1/2 activation (2-fold for 931HS2 cells and 3-fold for 606HS2 cells) and was associated with major changes in the pattern of Bim_EL_ expression (Figure 2B). Phosphorylated Bim_EL_ levels were increased in transduced HS2 cells compared to control HS2 cells. To further confirm the role of Shp2 activity in the phosphorylation of Bim_EL_, the catalytically-inactive Shp2^C459S^ mutant [19] was overexpressed in HS2 cells through infection with a retrovirus transducing Myc-tagged Shp2^C459S^. Overexpression of this mutant induced changes resembling to those induced by Shp2 depletion *i.e.* a disappearance of phosphorylated Bim_EL_, an increase in unphosphorylated Bim_EL_ and a major reduction in Erk1/2 activation associated with a marked cleavage of caspase 3 (Figure 2B). These observations indicate that Shp2 phosphatase activity controls Bim_EL_ expression in leukemic cells through its ability to induce Erk1/2 activation.

The PI3K/Akt pathway can also regulate Bim_EL_ expression. In particular, a loss of Akt activation can lead in turn to activation of FoxO3A transcription factor, resulting in an increased transcription of *Bim* [20]. However, no change in Bim_EL_ expression was detectable when HS2 cells were treated with the PI3K inhibitor NVP-BEZ235 (Figure 2C). Similarly, no change in Bim_EL_ expression was seen in HMC-1.2 cells following a treatment with the PI3K inhibitor NVP-BEZ235 (Figure 2C), whereas Akt phosphorylation was abolished (Figures 1C and 2C). Thereby, the expression of Bim was not influenced by the PI3K/Akt activity, a finding consistent with the absence of cross-talk between PI3K/Akt and Shp2/Mek/Erk signaling pathways in these cells [4]. Though Stat5 signaling did not contribute to apoptosis resistance of leukemic cells, we also examined whether Stat5 could modulate Bim expression. Bim expression was compared in HS2 cells infected either with a lentivirus encoding a short hairpin RNAs for Stat5 or a control non silencing shRNA. As shown in Figure 2D, Stat5 depletion did not affect Bim expression.

Altogether, these findings highlight the function of Shp2 as a major player in controlling Bim expression in the context of an oncogenic signaling.

### Knockdown of Bim Rescues HS2 Leukemic Cells from UO126-induced Apoptosis

**Figure 3:**
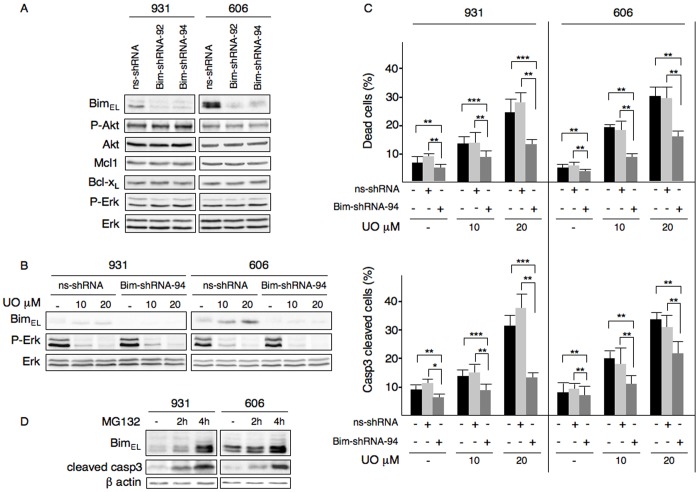
Knocking-down of Bim expression leads to attenuation of apoptosis induced by UO126 in HS2 cells. (**A**) Knocking-down of Bim in 606HS2 and 931HS2 cells. Whole cell extracts were immunoblotted using indicated antibodies. **(B)** Erk1/2 activation and Bim_EL_ expression in UO126 treated cells. Whole cell extracts from Bim-shRNA-94-HS2 cells or control ns-shRNA-HS2 cells treated or not (–) during 48hours with UO126 (10 or 20 µM) were immunoblotted using indicated antibodies. **(C)** For mortality analysis, cells were plated at 2×10^5^ cells/mL. Bim-shRNA-94-HS2 cells or control ns-shRNA-HS2 cells were treated or not (–) during 48hours with UO126 (10 or 20 µM) 24 h after lentiviral infection. The percentage of dead cells was evaluated by trypan blue exclusion. The percentage of apoptotic cells was determined by labeling the cells with cleaved caspase3-antibody and flow cytometry analysis. For both cell death and cell apoptosis, data are mean ±SEM (n = 3). Statistical differences from the value of the control (–) are indicated as follows: * *P*<0.05;** *P*<0.01; *** *P*<0.001 (Student *t* test). **(D)** Proteosomal degradation of Bim_EL_. Cells were treated or not (–) for 2 h or 4 h with MG132 (10 µM). Whole cell extracts were immunoblotted using indicated antibodies. Data are a representative experiment out of three.

To go further in the relevance of the down-regulation of Bim_EL_ in response to Erk1/2 activation, we studied whether extinction of Bim may overcome the mortality induced by Erk1/2 inactivation in HS2 cells. HS2 cells were infected with lentiviruses transducing independent Bim sh-RNAs (shRNAs 92 or 94). Both induced a major extinction in Bim expression (90 to 95%) without detectable alterations in the expression of Mcl-1, Bcl-x_L_, Erk1/2 and Akt (Figure 3A). The Bim-shRNA94-transduced 606HS2 and 931HS2 cells and control cells were treated during 48hours with UO126 at 10 µM or 20 µM. We controlled that Bim depletion was maintained during UO126 treatment (Figure 3B). At the dose of 10 µM UO126 corresponding to IC_50_ (as previously published [21]), a residual activation of Erk1/2 was detected, which was totally abolished when cells were treated with 20 µM UO126 (Figure 3B). Treatment of both ns-shRNA-HS2 cells and Bim-shRNA-94-HS2 cells with 20 µM UO126 resulted in a cell death that was more marked than after treatment with 10 µM UO126 (Figure 3C). This observation was consistent with the residual Erk1/2 activation seen with 10 µM of UO126. Remarkably, when cells were transduced with Bim shRNA (Figure 3B), the number of dead cells associated with UO126 treatment was reduced: 25% or 54% decrease with 10 µM or 20 µM UO126 in Bim-shRNA-94-931HS2 cells and 52% or 50% decrease with 10 µM or 20 µM UO126 in Bim-shRNA-94-606HS2 cells compared with ns-shRNA-HS2 cells.

To go further, the number of apoptotic cells as detected by flow cytometry analysis of active caspase-3 was determined in Bim-shRNA-94-HS2 and ns-shRNA-HS2 cells treated with 10 µM or 20 µM UO126 (Figure 3C). The percentage of apoptotic cells (cleaved caspase-3 positive) was significantly decreased compared to controls (40% or 65% decrease with 10 µM and 20 µM UO126 in 931HS2 cells; 43% or 34% decrease with 10 µM and 20 µM UO126 in 606HS2 cells). We observed similar results when experiments were conducted with Bim-shRNA-92-HS2 cells (data not shown). Thus, depletion of Bim did significantly overcome apoptosis induced by UO126 treatment indicating the contribution of Bim to drug-induced apoptosis.

Intriguingly, the level of both spontaneous apoptosis and UO126-induced apoptosis did not appear to be related to the expression level of Bim_EL_. Indeed, while 931 and 606 HS2 cells expressed different amount of Bim_EL_, the rate of spontaneous or UO126-induced apoptosis was quite similar (Figure 3C). To rule out the possibility that the high level of Bim_EL_ in 606HS2 cells expressing mutant Kit^D814Y^ reflected a cell line particularity, we analyzed Bim_EL_ expression in ten independent HS2 cell lines all carrying mutant Kit^D814Y^. Bim_EL_ expression level was reproducibly higher in these cells than that observed in 931HS2 cells expressing mutant Kit^D818Y^(data not shown). Thus, our data do not rule out that only a fraction of Bim_EL_ is involved in the apoptotic process in 606HS2 cells. However, the differences in Bim_EL_ expression might reflect differences in signaling downstream of Kit^D818Y^ in 931HS2 cells and Kit^D814Y^ in 606HS2 cells, producing different requirements for Bim during apoptosis.

### Bim Level is Regulated by a Post-translational Mechanism

The mechanisms for regulating Bim expression vary according to the apoptotic stimulus and the cell type. One mechanism initiated in response to Erk1/2 activation is the proteasomal degradation of the phosphorylated form of Bim_EL_ [17], [22]. To check this possibility, we treated HS2 cells with the proteasome inhibitor MG132 and monitored expression of Bim_EL_ by immunoblotting. Exposure of HS2 cells to MG132 (2 or 4 hours) resulted in a time-dependent increase of both phosphorylated and unphosphoryated Bim_EL_ forms (Figure 3D). Notably, a major increase in cleaved caspase-3 was detected paralleling the increase in Bim_EL_ amount in cells treated with MG132 for 4 hours. Altogether, these data show that proteosomal degradation is the main process leading to maintain a low level expression of Bim in the leukemic cells and that impeding Bim down-regulation is associated with apoptotic signals in the leukemic cells. This mode of Bim regulation is in agreement with the down-regulation of Bim caused by different oncogenic kinases in different malignant hemopathies, such as BCR-ABL in chronic myeloid leukemia [17], [23], Kit mutant in systemic mastocytosis [24] and JAK2^V617F^ in myeloproliferative disorders [25].

Bim is a BH3-only protein discovered for its ability to interact via the BH3 domain with the prosurvival proteins like Bcl2 or Mcl1 [16], [26], thereby being a direct antagonist of pro-survival proteins. This led to design BH3 mimetics compounds capable to neutralize the function of pro-survival proteins of Bcl2 family and usable in therapy. In hematological malignancies, BH3 mimetics are undergoing clinical trials [6]. HS2 cells are sensitive to Obatoclax [4], but resistant to ABT-737(data not shown). Obatoclax and ABT-737 are BH3 mimetics that differ in their ability to target pro-survival proteins. Obatoclax binds to all Bcl2 members (Bcl2, Bxl-x_L_, Bcl_W_, Mcl-1 and A1) [27] whereas ABT-737 fails to bind to Mcl-1 and A1 [28]. Since HS2 cells do not express A1, it is attractive to consider that Mcl-1 is the most likely interactor of Bim_EL_. Since the expression of Mcl-1 is not sensitive to inhibition of Erk1/2 and Kit in cells harboring mutant Kit^D814Y^ (606 HS2 cells) or Kit^D818Y^ (931 HS2 cells) (Figure 1A, 1B), the regulation of Bim_EL_ expression level appears as the key mechanism that creates the imbalance between prosurvival and proapoptotic activities in Kit-driven leukemia. This underscores that all strategies to mimic Bim activity are valuable therapeutic approaches to abolish the aberrant survival of leukemic cells. However, though Obatoclax is efficient to induce apoptosis in murine HS2 and human HMC-1.2 cells, Obatoclax alone is not sufficient for suppression of leukemic cells [4]. The association of Obatoclax with a PI3K inhibitor such as NVP-BEZ235 to reduce cell proliferation, is required for a complete eradication of leukemic growth. Our present data provide a rationale to this combination of drugs. They highlight that using BH3 mimetics in combination with PI3K inhibitors is a promising strategy to treat leukemia, especially in the case of an oncogenic signaling refractory to Tyrosine Kinase inhibitors.
